# Adapting the Trauma History Questionnaire for use in a population of homeless people with severe mental illness in Tamil Nadu, India: qualitative study

**DOI:** 10.1192/bjo.2021.952

**Published:** 2021-07-05

**Authors:** Andrew R. Gilmoor, Smriti Vallath, Ruth M. H. Peters, Denise van der Ben, Lauren Ng

**Affiliations:** Department of Science, Vrije Universiteit Amsterdam, The Netherlands; Department of Science, Vrije Universiteit Amsterdam, The Netherlands; Department of Psychology, Banyan Academy of Leadership in Mental Health, India; and The Banyan, India; Department of Science, Vrije Universiteit Amsterdam, The Netherlands; and Department of Global Health and Social Medicine, Harvard Medical School, USA; Department of Science, Vrije Universiteit Amsterdam, The Netherlands; Department of Psychiatry, Boston University School of Medicine, USA; and Department of Psychology, University of California Los Angeles, USA

**Keywords:** Trauma, post-traumatic stress disorder, qualitative research, transcultural psychiatry, rating scales

## Abstract

**Background:**

The Trauma History Questionnaire (THQ) is one of the most widely used traumatic event inventories, but its lack of validation makes it unsuitable for the millions of homeless people with severe mental illness in India, who are particularly vulnerable to trauma exposure.

**Aims:**

To translate and culturally adapt the THQ for use in a population of homeless people with severe mental illness in Tamil Nadu, India.

**Method:**

We used Herdman et al's model of cultural equivalence to conduct an in-depth qualitative assessment of the cultural validity of the THQ. Following several translations, conceptual, item, semantic and operational equivalence of the THQ was assessed through four focus groups with user-survivors (*n* = 20) and two focus groups with mental health professionals (*n* = 11).

**Results:**

Several adaptations, including the addition of 18 items about relationships, homelessness and mental illness, were necessary to improve cultural validity. Three items, such as rape, were removed for reasons of irrelevance or cultural insensitivity. Items like ‘adultery’ and ‘mental illness’ were reworded to ‘extramarital affair’ and ‘mental health problem’, respectively, to capture the cultural nuances of the Tamil language. Findings revealed a divergence in views on tool acceptability between user-survivors, who felt empowered to voice their experiences, and mental health professionals, who were concerned for patient well-being. Providing a sense of pride and autonomy, user-survivors preferred self-administration, whereas mental health professionals preferred rater administration.

**Conclusions:**

Culture significantly affects what types of events are considered traumatic, highlighting the importance of cultural validation of instruments for use in novel populations and settings.

Although most studies in the field of trauma relate to the psychological effect of potentially traumatic events (PTEs), such as post-traumatic stress disorder, fewer studies focus on the PTEs themselves. Commonly referred to as any event that is considered psychologically overwhelming for an individual and occurs outside ‘normal’ human experience,^1^ exposure to PTEs is fairly common globally.^2^ Although common, types and prevalence of PTEs differ greatly across populations and countries. Studies have indicated varying population prevalence of trauma exposure per country, ranging from 29% in Bulgaria to 83% in Peru.^2^ Although trauma exposure disproportionately affects populations of lower socioeconomic status,^3^ most epidemiological studies on trauma exposure focus on populations in high-income countries.^4^

## Trauma exposure in homeless people with severe mental illness

One population that is particularly vulnerable to trauma exposure is homeless people with severe mental illness (SMI). First, a wide range of PTEs have been linked to homelessness, including domestic violence, sexual abuse, physical abuse, war and natural disasters.^5,6^ Additionally, there is substantial evidence indicating that people with SMI are more susceptible to trauma exposure than the general population.^7^ For example, in the USA, a cohort of 275 patients with SMI had a 98% prevalence of lifetime exposure to traumatic experiences.^8^ Apart from high rates of reported sexual or physical abuse and exposure to interpersonal violence, people with SMI also report the development of SMI and the experience of hospitalisation as traumatic experiences.^9^ The complex nexus between homelessness and SMI make investigating trauma in this population with multiple vulnerabilities a matter of urgency.

## Homelessness and SMI in India

According to a 2011 census, the population of homeless individuals living in India was reported at approximately 1.8 million;^10,11^ however, this number is said to be a gross underestimation, with other records reporting numbers as high as 78 million.^10^ With up to 40% of homeless people reported to suffer from SMI,^12^ this accounts for a largely neglected subdivision of the population that remains particularly vulnerable to traumatic experiences. To determine the magnitude of trauma exposure in populations of homeless people with SMI in India, there is a need for the adaptation and development of tools to accurately identify PTEs in a manner that is both relevant and culturally sensitive to the needs of the target population.

## Trauma inventory

Originally developed for use in psychosocial research projects, the Trauma History Questionnaire (THQ) is one such tool that has been one of the most widely used traumatic event inventories available.^13^ The 25-item self-reported assessment examines lifetime exposure to PTEs grouped into three main domains: crime-related events (4 items), general disaster and trauma (13 items), physical and sexual experiences (7 items) and other events (1 item). Each item is prompted by a no/yes response, followed by the number of times and approximate age at which each event occurred, if a yes was indicated. Respondents are also prompted to specify details of the event and their relationship to those involved, where appropriate. The tool's use of neutral behavioural language and its structured no/yes response as opposed to open-question format has made it particularly useful for health researchers and care providers in diverse settings. The THQ is intended to gather information concerning the lifetime history of trauma exposure from general, community and clinical populations,^13^ and has been used in a diverse range of populations and settings, including trauma exposure in populations experiencing SMI,^14^ the impact of childhood trauma on psychosis in China,^15^ and trauma exposure in a rural primary care setting in South Africa.^16^

## Problem statement and study aims

Scholars have called into question the cross-cultural validity of the construct of trauma in settings outside of the original construct's Western origin, expressing that if the concept of trauma is intended to describe a situation that is outside the norm of human experience, then this norm is highly subjective to the cultural context in which trauma is being explored.^17^ As the DSM exclusively considers events to be traumatic if they are life-threatening or jeopardise one's physical integrity, there is ongoing debate as to whether this classification of a traumatic event is appropriate, or whether it underestimates the true extent of trauma exposure in different populations.^1,18^ Although there is record of the THQ's application in various cultural contexts, there is very limited published record of its cultural adaptation for use in different cultural contexts.^19^ Therefore, the aim of this study is to contribute to the applicability of trauma inventories in a population of homeless people with SMI in Tamil Nadu, India, by translating and culturally adapting the THQ for use in this population. Additionally, it explores the theoretical implications of the study's findings on the construct of trauma, sheds light on patient and provider perspectives on trauma assessment, and introduces the moral debate of asking culturally sensitive questions.

## Method

### Design

To determine the cross-cultural validity of the THQ for use in a population of homeless people with SMI in Tamil Nadu, India, a qualitative approach was taken, consisting of a number of translation exercises, focus group discussions and expert consultations. Several studies have demonstrated the added value of applying qualitative methods as a means to successfully culturally validate a variety of measurement instruments.^19,20^

### Approach to inquiry

This study takes both an emic and constructivist approach to the investigation of trauma in the context of homelessness and SMI in Tamil Nadu, India, which considers both group and individually influenced nuances that may shape the experience of trauma, respectively. First, several studies have highlighted the issue of applying methods for identifying and treating trauma that have been based on observations made in Europe and North America to low- and middle-income countries, where experiences are almost certainly shaped by local context.^18,21,22^ Inspired by this, the present study is informed by an emic approach, in which research efforts are focused on understanding local understanding and experiences of trauma, to better inform practice and research. Second, this study adopts a constructivist approach, which posits that the construct of reality is subjective and shaped by the norms, customs and values of the individual.^23^ In this context, a constructivist considers a traumatic experience to be subjective, and influenced by the sociocultural and political context in which the experience is had.^17^

### Researcher reflexivity

Author A.R.G. is a global mental health researcher currently pursuing his doctoral degree in The Netherlands. Although none of the research participants know the principal investigator personally, his extended presence and active participation in the daily happenings within the non-governmental organisation for up to 9 months before data collection made him a familiar face among participants. S.V. is a local, practicing clinical psychologist with 3 years of experience working with this specific population before data collection, and is well-versed in the local language. Her extensive theoretical and contextual knowledge, as well as experience, enriched the data collection process. Any potential sources of bias and data misinterpretation were extensively discussed among authors A.R.G., S.V. and D.v.d.B. during data collection and analysis.

### Setting

This study took place in the Kanchipuram and Chennai districts of Tamil Nadu, located on the south-eastern coast of India, along the Bay of Bengal. Attributed to its geographical positioning and low-resourced surrounding rural settlements, the city of Chennai has one of the highest population densities of urban homeless in India,^11^ with as many as 40 000 homeless individuals reported.^10^

### Participants and recruitment

Mental health professionals (MHPs) and user-survivors were recruited for participation in this study from within The Banyan network of care – a local, non-profit, non-governmental organisation, that caters to over 3000 homeless individuals and serves approximately 1 million people with mental health challenges across India, in a diverse network of mental health services. The term user-survivors is used to describe homeless people or those at risk of homelessness who have been diagnosed with SMI, and make use of The Banyan services in a long-term, short-term, in-patient or out-patient capacity. To account for potential differences among user-survivors, participants were recruited from a variety of The Banyan services. User-survivors who were intellectually disabled or perceived to be too symptomatic to comprehend the instructions of the focus group discussions were excluded from participation.

To gain a broad range of perspectives, a diverse sample of MHPs of varying mental health professions, including psychology, psychiatry and social work, were recruited. Most MHPs were fluent in both Tamil and English, and had been mental health practitioners within The Banyan for a minimum of 2 years.

### Data collection

Data collection took place in three different phases of work.

#### Phase 1: tool adaptation

Phase 1 was part of a larger project, the findings of which are published elsewhere.^21^ In short, a series of free-listing exercises and in-depth interviews with 26 user-survivors were used to identify PTEs that were relevant to people who were homeless, or at risk of homelessness, and living with mental illness in Tamil Nadu. User-survivors were asked to describe what they understood to be trauma (*adirchi* in Tamil), and list as many events as possible that they had either experienced or considered to be traumatic. Analysis of the interview transcripts generated a composite list of events perceived to be traumatic by user-survivors. The first author then cross-referenced this list to the 24 existing PTEs listed in the original THQ. Items that were not found to be relevant to the local context were removed from the original scale, and additional items thought to be relevant were added.

#### Phase 2: translation

The tool was translated using the translation and back-translation approach as described by Gudmundsson (2009).^24^ The adapted THQ was first translated into Tamil (T1) by an English–Tamil bilingual professional medical transcriptionist, who has extensive experience in translation and transcription work in both languages. Using four blinded translators of varying professional backgrounds, T1 was then independently back-translated into English (BT1, BT2, BT3 and BT4). A multidisciplinary team of experts, consisting of a bilingual clinical psychologist, a bilingual social worker, and English-speaking principal investigator and co-researcher, was then consulted and incorporated the feedback into a newly adapted THQ (THQ^T2^).

#### Phase 3: cognitive testing and cross-cultural adaptation

Next, a series of six focus group discussions were used to test and further develop the THQ^T2^ by evaluating how the target population understands and responds to the questionnaire. This qualitative approach was employed to ensure an in-depth exploration of participants’ views on specific aspects of the questionnaire. Four focus group discussions consisted of five, seven, five and three female user-survivors, respectively. At the time of recruitment, there were no potential male user-survivor participants that met the inclusion criteria of illness stabilisation and reduction in symptoms since beginning treatment at The Banyan. Two additional focus group discussions consisted of MHPs, with one group recruited for their language expertise as first-language Tamil speakers (*n* = 5), and another for their experience in care provision within The Banyan (*n* = 6). Details of the study sample recruited for the focus group discussions are presented in Table 1.

**Table 1:** Study sample characteristics

Focus group discussion	Population	Composition	The Banyan project site
1	User-survivors	5 women	CGH
2	User-survivors	7 women	ECRC
3	User-survivors	5 women	ECRC
4	User-survivors	3 women	HSS
5	Language experts	4 women 1 man	Combination
6	Mental health professionals	5 women 1 man	Combination

CGH, Clustered Group Home; ECRC, Emergency Care and Recovery Center; HSS, Housing with Supportive Services.

The design of the focus group discussions were based on Herdman et al's model for assessing cross-cultural equivalence in health-related quality of life questionnaires,^25^ which draws from extensive research in cross-cultural work on health-related quality-of-life studies. Based on six aspects of equivalence needed to be achieved to determine a tool's cross-cultural validity (conceptual equivalence, semantic equivalence, item equivalence, operational equivalence, measurement equivalence and functional equivalence), this framework has been successfully applied in a number of other cross-cultural tool development studies.^19,26–28^ Because of the subjective nature of trauma and the THQ's function as an inventory, the scope of this study was limited to the first four types of equivalences. The definition and operationalisation of the types of equivalence used in this study are presented in Table 2.

**Table 2:** Description of the different categories of equivalence used in this study, as described by Herdman et al^25^

Term	Definition and operationalisation	Measures/indicators
Conceptual equivalence	Refers to whether the scale and its different domains are representative of how traumatic experiences are conceptualised in both the original and target populations' cultures	What domains are relevant and irrelevant in the original THQ for the conceptualisation of trauma in the target population?
Item equivalence	Refers to whether user-survivors identify the individual items (traumatic experiences) listed in the THQ^T2^ as a relevant traumatic event, and representative of its assigned domain	Relevance: are the items conceptually relevant as types of traumatic experiences to the target population? Acceptability: is it culturally acceptable to ask certain items on the list?
Semantic equivalence	Refers to whether items within the THQ^T2^ are translated into the local language (Tamil) in a way that captures similar meaning, with the same effect in the target population as intended by the original THQ	Referential: does the translated word refer to the same thing as the original? Connotative: does the translated word produce the same emotive effect as the original? Register: is the level of language used appropriate for the target population?
Operational equivalence	Refers to whether the THQ^T2^ can be administered to user-survivors in a similar format as intended by the original THQ, or whether changes to the form of administration do not affect the outcomes of the inventory	Mode of administration, questionnaire format, measurement methods

THQ, Trauma History Questionnaire; THQ^T2^, second revision of the adapted Trauma History Questionnaire.

All focus group discussion participants were first instructed to complete the THQ^T2^. Next, a series of questions reflecting Herdman et al's model^25^ were addressed during the group discussion. Conceptual and item equivalence were addressed by examining which of the seven domains and 37 items of the THQ^T2^ were relevant to user-survivors. The group also discussed what other traumas should be listed, and what items they felt were either inappropriate or not applicable. Semantic equivalence was evaluated by cognitive assessment. Participants were instructed to read each item and then share its interpreted meaning with the rest of the group. In this way, the readability and clarity of each item and the overall tool was assessed. Operational equivalence was addressed by discussing advantages and disadvantages of self-administration and rater administration. Any errors, misunderstood item/terms or unclear elements of the THQ^T2^ were also addressed.

### Finalising the THQ

All focus group discussion notes and transcripts were examined by a multidisciplinary team of professionals, consisting of a global health researcher, a clinical psychologist and a social worker. Using this feedback, the THQ^T2^ was once again modified, translated and back-translated to develop the THQ^T3^. A final team of experts, consisting of two clinical psychologists, a social worker, a counselling therapist, a psychiatrist and the principal investigator, reviewed the latest version. Final recommendations were consolidated and incorporated into a new version of the adapted THQ: the THQ for Multiple Vulnerabilities (THQ^MV^), ready to be piloted (see Supplementary Appendices 1 and 2 available at https://doi.org/10.1192/bjo.2021.952).

### Analysis

A directed-content analysis approach, as described by Hsieh and Shannon,^29^ was undertaken for analysis. First, all digitally recorded focus group discussions were transcribed verbatim and translated to English. All transcripts, notes and further documentation of the changes made to the original THQ, THQ^T1^, THQ^T2^ and THQ^T3^ were studied for familiarisation of the data. Using Herdman et al's model for cultural equivalence,^25^ a deductive approach was taken for analysis. Additional codes were created to account for important aspects of the data that did not fit any of the predefined codes. Excerpts tagged with the same codes were consolidated and examined for higher-level analysis.

For quality assurance, all transcripts were independently coded and analysed by authors A.R.G. and D.v.d.B. To test for inter-coder reliability, two randomly selected transcripts were additionally coded and analysed by author S.V. and compared with the coded transcripts of A.R.G. The number of agreed upon codes as a percentage of the total number of codes identified was used to determine inter-coder reliability, for which a score of 75% agreement was deemed as acceptable. Any divergence in interpretation of the data was discussed until consensus was achieved.

### Ethics statement

The authors assert that all procedures contributing to this work comply with the ethical standards of the relevant national and institutional committees on human experimentation and with the Helsinki Declaration of 1975, as revised in 2008. All procedures involving human participants were approved by the Dutch Science Committee of the Institute for Health and Care Research (EMGO+) (approval number WC2015-049 HZ) and The Banyan Internal Ethics Committee in Chennai.

### Consent statement

Verbal informed consent was obtained from all patients. Verbal consent was witnessed and formally recorded by authors A.R.G. and S.V..

## Results

Findings suggest that extensive modifications to the THQ were needed to achieve cultural equivalence for use in the target population.

### Conceptual equivalence

#### Trauma domains

Based on the free-listing exercises conducted, user-survivors recognised three domains of traumatic experiences – namely, ‘mental health experiences’, ‘homeless experiences’ and ‘relationship issues’ – in addition to the four domains listed in the original THQ. For mental health experiences, user-survivors considered the development of their mental illness, associated stigma and negative health experiences as conceivably traumatic (Table 3, quote 1). Experiences of a lack of basic necessities, a lack of security, and abandonment were homeless experiences commonly reported as traumatic by user-survivors (Table 3, quote 2). Finally, events such as divorce or abandonment by a family member were commonly considered plausibly traumatic experiences related to relationship issues (Table 3, quotes 3 and 4). Feelings of a lack of representation of these three domains, and particularly of homeless experiences, were shared in the focus group discussion conducted with MHPs (Table 3, quote 5). Based on the insights and feedback of both user-survivor and MHP groups, the additional trauma domains of mental health experiences, homeless experiences and relationship issues were added to the THQ.

**Table 3:** Additional trauma domains as recommended by participants

QuoteID	Trauma domain	Quote	Participant (gender, age, diagnosis)
1	Mental health experiences	‘If the people around you don't understand…and they put [you] in an institution, [you] get trauma because there is no place for [you] at home and [you] have been put here [in an institution] and it is worse here than there’	Female, 75 years, schizophrenia
2	Homeless experiences	‘When you need food and other things…and there is no way of getting and you don't know any way out then that is traumatic’	Female, 75 years, schizophrenia
3	Relationship issues	‘A separation of husband and wife relationship. That can be traumatic’	Female, 43 years, schizophrenia
4	Relationship issues	‘I had a child [who] told everyone that they were getting married, but they didn't inform me. That was very traumatic to me’	Female, 45 years, bipolar affective disorder
5	All domains	‘In terms of all the categories that you mention, you really diminish the relevance of the homeless concept itself. It is not visible at all. It is reflected in a way where it looks like it is not so big’	Participant 1, focus group discussion 6

### Item equivalence

#### Item relevance

In total, three items were dropped and 21 items were added to the THQ to achieve item equivalence in the adapted THQ^T2^ (Table 4). Two of the items deemed irrelevant to the target population dealt with exposure to hazardous chemicals and engagement in politically driven conflict, as no user-survivors reported such incidents occurring in this population. A third item, addressing the culturally sensitive topic of rape, was deemed too intrusive in its current form.

**Table 4:** Overview of items added and removed from the Trauma History Questionnaire for item equivalence

Item number	Item question
Added items	
General	
Item 16	Have you ever witnessed a friend, relative, parent or spouse suffer from a serious or life-threatening illness?
Item 18	Have you ever experienced a sudden financial loss?
Relationship issues	
Item 19	Have you ever been the victim of alcohol or substance abuse?
Item 22	Has your partner, husband or wife ever had extramarital relations?
Item 23	Have you ever been separated divorced? If yes, please specify who.
Item 24	Has your partner, husband or wife ever taken up a second spouse?
Item 20	Has shame ever been brought upon your family due to some reason?
Item 21	Have you ever felt alienated or abandoned by a husband, wife, family member, or close friend?
Homeless experiences	
Item 32	Have you ever gone without shelter? If yes, what was the longest period you've gone without shelter?
Item 33	Have you ever gone without food? If yes, what was the longest period you've gone without food?
Item 34	Have you ever gone without water? If yes, what was the longest period you've gone without water?
Item 35	Have you ever gone without proper hygiene? If yes, what was the longest period you've been without proper hygiene?
Item 36	Have you ever been without clothes? If yes, what was the longest period you've been without proper clothes?
Item 31	Have you ever experienced a loss of status?
Item 37	Have you ever felt ostracized by society?
Mental health experiences	
Item 40A	Have you ever developed a mental illness?
Item 40B	If yes, have you ever been stigmatized due to your mental illness
Item 38	Have you ever been troubled or stressed?
Item 39	Have you ever had a mental condition?
Item 42	Have you ever gone without care for your mental condition? If yes, please specify for how long
Item 41	Have you ever been institutionalized?
Removed items	
General	
Item 8	Have you ever been exposed to dangerous chemicals or radioactivity that might threaten your health?
Item 17	Have you ever had to engage in combat while in military service in an official or unofficial war zone?
Physical and sexual experiences	
Item 20	Other than incidents mentioned in Questions 18 and 19, have there been any other situations in which another person tried to force you to have unwanted sexual contact?

The 21 added items mainly contribute to the added trauma domains of homeless experiences (*n* = 7), mental health experiences (*n* = 6) and relationship issues (*n* = 6). Two items were additionally added to the domain of general disasters and trauma. There was unanimous agreement across all stakeholder groups that the types of traumatic experiences listed in the THQ^T2^ were indeed relevant to the intended target population.

Although universally applicable according to the majority of respondents, there was general consensus among MHPs that items could be further specified to specifically fit the Indian context (Table 5, quotes 1 and 2).

**Table 5:** Overview of item amendment for cultural relevance

Item number	THQ^T4^	Function	THQ^T2^
1	e.g. Property or money	Added to	Has anyone tried to take something from you
6	Cyclone, tsunami	Replaced	Tornado, hurricane
18	Loss in livelihood	Added to	Financial loss
21	Acid, machete, kerosene	Replaced	Gun, knife
26	Separated	Added to	Divorce
35	Received care	Added to	Institutionalized

THQ^T4^, fourth revision of the adapted Trauma History Questionnaire; THQ^T2^, second revision of the adapted Trauma History Questionnaire.

#### Tool and item acceptability

Although there were some instances of rehashing some negative memories that were emotionally arousing, the general reception and acceptability of the THQ^T2^ was positive. User-survivors expressed that the inventory provided a form of release, providing a sense of relief and even joy (Table 6, quotes 3 and 4).

**Table 6:** Item equivalence and acceptability

QuoteID		Quote	Participant
1	Item equivalence	[the] words can be [more] specific I think. For example, if you use words like ‘gun’ in our context, threatening with a gun is a very uncommon thing, it is more a Western concept. Probably you can be threatened with acid [or] kerosene … I think in a particular culture and context also, you [need to make these] considerations	Participant 1, FGD6
2	Item equivalence	‘In the Indian context, you may not be [institutionalised], but you can get support, [or] coping from the community. That is not captured here. Because your [tool has a] very biomedical oriented approach. So, I'm saying you can also find out, for example, “my family would withhold support”, so you need to capture that’	Participant 1, expert consultation
3	Tool acceptability	‘It was a nice experience. I felt free and open. I recollected my childhood memories and wrote down everything. I felt like I opened my heart’	Participant 3, FGD1
4	Tool acceptability	‘I let out my feelings, so I feel happy. Whatever sadness I had in mind I let it out’	Participant 1, FGD4
5	Item acceptability	‘It is wrong to ask if a person is raped’	Participant 2, FGD1
6	Item acceptability	‘Yes, you should not ask it’	Participant 4, FGD2
7	Item acceptability	‘It is really hurting’	Participant 2, FGD2
8	Tool/item acceptability	‘Knowing who it is, is it going to [add anything]? … Because if something like that would happen in my life, [for instance], if it was my father, how [could] I list it down?’	Participant 1, FGD6

FGD, focus group discussion.

With one exception, all items were deemed acceptable by the user-survivor participants. However, it was unanimously agreed that item 20 (‘Has anyone ever touched private parts of your body, or made you touch theirs, under force or threat?’) was considered too offensive and not socially acceptable to be asked in its current form (Table 6, quotes 5–7). This item was later adapted in the subsequent version of the THQ^T4^ to ‘Has anyone ever touched you or made you touch them inappropriately, under force or threat?’. There were a number of MHPs who felt that specifying the relationship between victim and perpetrator in a number of the items listed was invasive, and questioned its added relevance to the inventory (Table 6, quote 8). However, these views were not shared by the target population, and so were not incorporated.

### Semantic equivalence

#### Referential equivalence

To achieve referential equivalence, several recommendations were made by the language experts (Table 7). The term *bodhai pural*, for example, specifically translates to ‘drug addiction’ in Tamil, excluding other forms of possible addiction. This was later changed to ‘*bodhai sambandham adimai*’, which translates to substance-related addiction, to capture the intended referential meaning of substance misuse. Interestingly, no Tamil translation could be found for the term ‘stigma’. Based on deliberations with the language experts, the Tamil word for shame (*kalangam)* was used to refer to stigma, as it was found to be the closest match for the semantic meaning of stigma.

**Table 7:** Semantic equivalence

Item number	Item	Original Tamil translation	Implication	Recommended Tamil translation	English back-translation	Type of semantic equivalence
17	Have you ever received news of a **serious injury**, life-threatening illness or unexpected death of someone close to you?	*Mosamanna kaayangalal*	Translates to ‘bad’ injury	*Balatha kayam*	Serious injury	Referential
18	Have you ever experienced a sudden **financial loss**?	*Nidhi izhapu*	Translated to ‘loss of funds’	*Porulaadhara izhapu*	Financial loss	Referential
22	Has anyone ever repeatedly bullied, humiliated, tried to intimidate and/or **succeeded** in intimidating you?	*Vetri petraargala*	Implies victory	No Tamil translation	Rephrased to ‘Has anyone intimidated you?’	Connotative
22	Has anyone ever repeatedly bullied, humiliated, tried to **intimidate** and/or succeeded in intimidating you?	*Mirata*	Translates to threaten	*Nerukudhal*	Intimidated	Referential
28	Has your husband or wife ever committed **adultery**?	*Vibachaarathil*	Directly translates to prostitution: implies that it is an illegal act	*Thirumanairthuku apparpathu uruvu*	Extramarital affair	Connotative
25	Did your husband or wife ever suffer from an alcohol or **substance abuse** problem?	*Bodhai pural*	Drugs	*Bodhai sambandham adimai*	Substance-related addiction	Referential
38	Have you ever developed a **mental illness**?	*Mana noyinaal*	Mental illness	*Mana nala badhipu*	Mental health problem	Connotative
38	Have you ever been **stigmatized** due to your mental illness?	*No translation*	-	*Kalangam*	Shame	Referential

Bolding in the item question indicates the specific word/phrase that was altered.

#### Connotative equivalence

A few adaptations for connotative equivalence were also necessary (Table 7). Both user-survivors and MHPs indicated that the term ‘adultery’ has a very specific negative connotation, as it directly translates to prostitution (*vibachaarathil*). After several deliberations, the term was replaced with ‘extramarital affair’ (*thirumanairthuku apparpathu kuruvu*) in an effort to maintain scale neutrality. The item ‘Have you ever developed a mental illness?’, although grammatically correct, was understood to be too confrontational because of the negative connotation of ‘illness’. As recommended, the term mental illness (*mana noyinaal)* was rephrased as mental health problem (*mana nala badhipu*).

### Operational equivalence

#### Mode of administration

The THQ^T2^ could only be self-administered by 5 out of the 20 user-survivors who participated in the initial pilot. The remaining 15 were rater-administered, either because they were unable to read or write in Tamil, or were physically impaired. Despite the need for assistance, all user-survivors in the pilot unanimously agreed that they preferred to complete the inventory on their own, reporting that it allowed them to exercise their individual autonomy and gave them a sense of pride and empowerment (Table 8, quote 1). Contrasted to user-survivors’ views, several MHPs firmly believed that, because of their condition and to eliminate bias and maintain accuracy, the adapted THQ should be strictly rater-administered (Table 8, quote 2). Other MHPs were less adamant on this issue. Although they generally believed that the inventory should not be entirely self-administered, they did acknowledge user-survivors’ appeal for autonomy. A compromise was decided in which a rater should be present for questions and assistance.

**Table 8:** Operational equivalence

QuoteID	Theme	Quote	Participant
1	Mode of administration	‘It was prestigious for me to write the answers for [the inventory]’	Participant 2, FGD2
2	Mode of administration	‘It's better to ask them and then see how they respond, so that we know whether they answer well or [if] they [answer] randomly’	Participant 2, expert consultation
3	Inventory format	‘The questions could maybe start with something that is not that intrusive. Especially if you look at the physical, sexual and also the martial experiences…it would be very intrusive for a person who has gone through trauma to start with a question about adultery or…rape’	Participant 2, FGD6
4	Measurement of trauma	‘They do not have the thinking ability to fill so many questions’	Participant 3, FGD1
5	Measurement of trauma	‘They will not be able to fill it…It will be too long for them’	Participant 4, FGD1
6	Measurement of trauma	‘A hierarchy of seriousness would be good … For instance, if you would do EMDR, you would first take either the most serious case or the most recent one. And it would be good for both the therapist and the client to know what is important to focus on’	Participant 3, Expert consultation

FGD, focus group discussion; EMDR, Eye Movement Desensitization and Reprocessing.

#### Inventory format

Overall, user-survivors and MHPs were satisfied with the format of the inventory. Several MHPs, however, felt that the present order in which the trauma domains and items were presented was too intrusive (Table 8, quote 3). For user-survivors to feel more comfortable and build up a tolerance to the potential offensiveness of the items addressed, we changed the order of presentation of domains by moving ‘physical and sexual experiences’ to after ‘relationship issues’, starting with items that are perceived as less intrusive, such as shame and alienation, to the more intrusive items of physical abuse, humiliation and sexual assault.

#### Measurement of trauma

Despite the need for initial clarification of the instructions, user-survivors were able to successfully meet the requirements of measuring trauma as prescribed by the original THQ. They were able to indicate whether they had experienced PTEs in the THQ^T2^ and were able to identify the number of times and age at which the traumatic event has occurred. A few MHP respondents, however, questioned the accuracy with which user-survivors are capable of achieving the latter. Additionally, user-survivors unanimously felt that if administered to symptomatic patients with mental illness, they would not be able to comprehend the inventory or endure its entire duration (Table 8, quotes 4 and 5).

In addition to the measures of trauma prescribed by the original THQ, MHPs reported wanting a rating of trauma severity, where user-survivors would be instructed to rate their experience of trauma from 1 to 5, in terms of distress experienced. MHPs felt that this was a contextual adaptation that was needed to allow practitioners to prioritise specific events for intervention in instances where multiple traumas are evident.

## Discussion

The findings of this study reveal three major points of reflection, which are described below.

### The construct of trauma has both generic and culturally specific elements

Our study reveals that in this particular population, there are aspects of trauma that are recognised as universal across all contexts and those that are unique to the context of homelessness and SMI in Tamil Nadu, India. Starting with generic elements, results indicate that the three trauma domains (crime-related events, general disasters and trauma, and physical and sexual experiences) represented in the original THQ were all recognised by the target population as traumatic types of events. These trauma domains, which are based on a model of eight generic stressor dimensions of trauma by Green,^30^ cover a wide range of events, primarily chosen because they have been historically considered important by clinicians and clinical researchers.

On the other hand, the items on the original THQ do not fully represent the experiences and perceptions of the target population, as it fails to capture experiences specific to homelessness, SMI and relationship issues – experiences regarded as conceivably traumatic in this population. Although PTEs are outside the realm of normal human experience, culture dictates what is normal and what is abnormal. Taking the example of the item divorce: with 40% of all marriages ending in divorce,^31^ the dissolution of marriage is a fairly normalised event in the USA, but, despite being on the rise in India in recent years, divorce rates are comparatively low at 0.24%, as reported in a 2011 census.^32^ Divorce in India is conceivably distressing, particularly for women, who may experience a substantial decline in standard of living and are often subjected to disapproval from family members and the community.^33^ Events such as rejection and abandonment, again, are conceivably distressing to anyone experiencing it; however, in a collectivist society such as in India, where social cohesion and interdependence are not only the norm, but also a lifeline for many households,^34^ these experiences can have devastating effects on the well-being and psyche of many individuals.

### There is significant divergence in views on tool acceptability between user-survivors and MHPs

It appears that MHPs and user-survivors have very different views on the acceptability of the THQ and its usefulness in clinical practice. Results revealed user-survivors to be more receptive of the adapted THQ, whereas MHPs remained critical. On the one hand, MHPs view the use of such trauma inventories in clinical practice as an impersonal and insensitive approach to obtaining the trauma histories of their patients. Contrarily, findings also show that user-survivors endorse the use of such inventories, feeling that their experiences have been recognised and their impact acknowledged. These findings on user-survivors’ experiences with tool administration are consistent with previous studies, such as that conducted by Carlson et al in a population of 223 psychiatric in-patients,^35^ but available literature on this topic is scarce.

Resistance to tool implementation by MHPs can be because of a number of reasons. First, MHPs perceptions that administering the adapted THQ may be distressing to their patients reveal a level of protectiveness over their well-being. However, overprotection can be a sign of underestimating patient capabilities and a hindrance to care. Several studies illustrate this divergence between patient and MHP.^36^ These paternalistic practices of care remain prevalent in Indian healthcare settings.^37^

### Balancing custom with necessity

Perhaps the most evident and controversial dilemma in the THQ cultural adaptation process is finding a balance between asking the necessary questions and remaining sensitive to the customs of the culture. From the results, both user-survivors, and in particular MHPs, were resistant to the inclusion of rape in the trauma inventory because they felt it was too intrusive and emotionally arousing. Historically, the subject of rape has been a largely taboo topic in Indian society. At an estimated 8.5%, the prevalence of rape in India is considered relatively low compared with other countries; however, with only 1% of victims of sexual violence reporting their crimes, this is a gross underestimation of the reality.^38^ Rape and other forms of sexual violence against women remain a taboo topic because not only does it result in physical harm of the victim, but it also disgraces and tarnishes the victim's psyche with devastating effect.^39^ With family members reported to be the most common perpetrators of sexual violence against women,^40^ the social implications and associated stigma of reporting such cases are even higher. In light of the current increased visibility of the issue, and dramatic upsurge in cases of sexual violence against women being reported, we as researchers are faced with the dilemma of either providing a means to shed light on this highly relevant issue, or comply with social customs and further fuel the stigma and lack of awareness by discounting the problem.

### Study limitations

Some study limitations must be noted. Our exclusion criteria limit our findings to the perspectives of user-survivors who were deemed cognitively abled and minimally symptomatic for participation in the focus group discussions. As the male user-survivors we attempted to recruit for this study were too symptomatic to participate, the feedback on the THQ was limited to the perspectives of female user-survivors of the target population. We suspect that although the items within the THQ^MV^ cover a range of PTEs that are relevant to both male and female user-survivors, differences in gender norms may dictate how comfortable some male respondents may feel in disclosing the occurrence of specific events.

### Further research

We recommend further research to contribute to the development of trauma inventories for use in populations of homeless people with SMI. Although the focus group discussions with user-survivors have illustrated their capability of taking the inventory, the consistency of their responses over time or with different raters has yet to be determined. Further research to test the psychometric properties, specifically test–retest and interrater reliability of the adapted THQ, is required. Further piloting with male user-survivors would be necessary to determine any gender-related differences in perspectives on the usability and acceptability of the THQ^MV^. Additionally, to understand potential differences in item response as a result of latent attributes such as gender, we recommend applying item response theory for further analysis.^41^ With the addition of 21 new items to the inventory, a closer examination of its feasibility in practice is necessary to minimise respondent fatigue. Finally, further piloting the THQ^MV^ on patients with varying cognitive ability and symptom levels is required to investigate the true extent of the THQ^MV^'s utility in patients with SMI. This would allow us to understand the full spectrum of patients capable of undergoing a trauma inventory, and at what stage in their recovery it would be most feasible for administration of the tool.

### Study implications and contribution

This study adds to the understanding of trauma as a valid construct in Indian populations, using qualitative means. At the same time, it reiterates Herdman et al's universalist approach to cross-cultural psychology.^20^ Although there are basic assumptions of what constitutes trauma that are recognised worldwide, culture significantly affects what types of events are considered traumatic, the severity of the impact and how it is addressed. We therefore recommend future studies use a universalist approach when validating screening tools for trauma. Although we expect that the applicability of the THQ^MV^ expands beyond homeless populations with SMI in Tamil Nadu, and is similarly relevant to other parts of the South Asia region, we recommend pre-explorative investigation of the needs and experiences of any target population, to ensure relevance and acceptability.

## Data Availability

Anonymised data are available from the corresponding author, A.R.G., upon reasonable request, following formalised agreements made between the authors and interested parties.

## Supplementary material

For supplementary material accompanying this paper visit http://dx.doi.org/10.1192/bjo.2021.952. click here to view supplementary material

## References

[1] Briere J, Scott C. Principles of Trauma Therapy: A Guide to Symptoms, Evaluation, and Treatment. Sage Publishing, 2006.

[2] Benjet C, Bromet E, Karam EG, Kessler RC, Mclaughlin KA, Ruscio AM, The epidemiology of traumatic event exposure worldwide: results from the World Mental Health Survey Consortium. Psychol Med 2016; 46: 327–43. 26511595 10.1017/S0033291715001981 PMC4869975

[3] Jarl J, Cantor-Graae E, Chak T, Sunbaunat K, Larsson CA. Trauma and poor mental health in relation to economic status: the case of Cambodia 35 years later. PLoS One 2015; 10(8): e0136410. 26301591 10.1371/journal.pone.0136410 PMC4547808

[4] Kilpatrick DG, Resnick HS, Milanak ME, Miller MW, Keyes KM, Friedman MJ. National estimates of exposure to traumatic events and PTSD prevalence using DSM-IV and DSM-5 criteria. J Trauma Stress 2013; 26(5): 537–47. 24151000 10.1002/jts.21848 PMC4096796

[5] Hopper E, Bassuk E, Olivet J. Shelter from the storm: trauma-informed care in homelessness services settings. Open Health Serv Policy J 2009; 2(617): 131–51.

[6] Kim MM, Ford JD, Howard DL, Bradford DW. Assessing trauma, substance abuse, and mental health in a sample of homeless men. Health Soc Work 2010; 35(1): 39–48. 20218452 10.1093/hsw/35.1.39

[7] Grubaugh AL, Zinzow HM, Paul L, Egede LE, Frueh BC. Trauma exposure and posttraumatic stress disorder in adults with severe mental illness: a critical review. Clin Psychol Rev 2011; 31(6): 883–99. 21596012 10.1016/j.cpr.2011.04.003 PMC3148295

[8] Mueser KT, Trumbetta SL, Rosenberg SD, Vidaver R, Goodman LB, Osher FC, Trauma and posttraumatic stress disorder in severe mental illness. J Consult Clin Psychol 1998; 66(3): 493–9. 9642887 10.1037//0022-006x.66.3.493

[9] Mueser KT, Rosenberg SD, Goodman LA, Trumbetta SL. Trauma, PTSD, and the course of severe mental illness: an interactive model. Schizophr Res 2002; 53(1–2): 123–43. 11728845 10.1016/s0920-9964(01)00173-6

[10] Sattar S. Homelessness in India. Shelter 2014; 15(1): 9–14.

[11] Kumuda D. Homeless population in India: a study. Glob J Res Anal 2014; 3(8): 54–5.

[12] Toro PA. Toward an international understanding of homelessness. J Soc Issues 2007; 63(3): 461–81.

[13] Hooper LM, Stockton P, Krupnick JL, Green BL. Development, use, and psychometric properties of the Trauma History Questionnaire. J Loss Trauma 2011; 16(3): 258–83.

[14] Shannon C, Maguire C, Anderson J, Meenagh C, Mulholland C. Enquiring about traumatic experiences in bipolar disorder: a case note and self- report comparison. J Affect Disord 2011; 133(1–2): 352–5. 21596441 10.1016/j.jad.2011.04.022

[15] Sun M, Zhang W, Guo R, Hu A, Li Y, Mwansisya TE, Psychotic-like experiences and correlation with childhood trauma and other socio-demographic factors: a cross-sectional survey in adolescence and early adulthood in China. Psychiatry Res 2017; 255: 272–7. 28595150 10.1016/j.psychres.2017.03.059

[16] Peltzer K, Seakamela MJ, Manganye L, Mamiane KG, Motsei MS, Mathebula TTM. Trauma and posttraumatic stress disorder in a rural primary care population in South Africa. Psychol Rep 2007; 100(suppl 3): 1115–20. 17886497 10.2466/pr0.100.4.1115-1120

[17] Patel V. Culture and the mental health consequences of trauma. Indian J Soc Work 2000; 61(4): 626–30.

[18] Jones LK, Cureton JL. Trauma redefined in the DSM-5: rationale and implications for counseling practice. Prof Couns Res Pract 2014; 4(3): 257.

[19] Fiszman A, Cabizuca M, Lanfredi C, Figueira I. The cross-cultural adaptation to Portuguese of the Trauma History Questionnaire to identify traumatic experiences. Rev Bras Psiquiatr 2005; 27(1): 63–6. 15867986 10.1590/s1516-44462005000100014

[20] Herdman M, Fox-Rushby J, Badia X. “Equivalence” and the translation and adaptation of health-related quality of life questionnaires. Qual Life Res 1997; 6(3): 237–47. 9226981 10.1023/a:1026410721664

[21] Gilmoor A, Vallath S, Regeer B, Bunders J. “If somebody could just understand what I am going through, it would make all the difference”: conceptualizations of trauma in homeless populations experiencing severe mental illness. Transcult Psychiatry 2020; 57(3): 455–67. 32148189 10.1177/1363461520909613 PMC7263042

[22] Rasmussen A, Keatley E, Joscelyne A. Posttraumatic stress in emergency settings outside North America and Europe: a review of the emic literature. Soc Sci Med 2014; 109: 44–54. 24698712 10.1016/j.socscimed.2014.03.015 PMC4070307

[23] Schwandt T. Constructivist, interpretivist approaches to human inquiry. In Handbook of Qualitative Research (eds N Denzin, Y Lincoln): 118–37. Sage Publishing, 1994.

[24] Gudmundsson E. Guidelines for translating and adapting psychological instruments. Nord Psychol 2009; 61(2): 29–45.

[25] Herdman M, Fox-Rushby J, Badia X. A model of equivalence in the cultural adaptation of HRQoL instruments: the universalist approach. Qual Life Res 1998; 7: 323–35. 9610216 10.1023/a:1024985930536

[26] Dadun PR, Van Brakel WH, Lusli M, Damayanti R, Bunders JF. Cultural validation of a new instrument to measure leprosy-related stigma: the SARI Stigma Scale. Lepr Rev 2017; 88(1): 23–42. 30188086

[27] Peters RMH, Dadun, Van Brakel WH, Zweekhorst MBM, Damayanti R, Bunders JFG, The cultural validation of two scales to assess social stigma in leprosy. PLoS Negl Trop Dis 2014; 8(11): e3274. 10.1371/journal.pntd.0003274 PMC4222778 25376007

[28] Stevelink SAM, van Brakel WH, Augustine V. Stigma and social participation in Southern India: differences and commonalities among persons affected by leprosy and persons living with HIV/AIDS. Psychol Health Med 2011; 16(6): 695–707. 21391136 10.1080/13548506.2011.555945

[29] Hsieh H-F, Shannon SE. Three approaches to qualitative content analysis background on the development of content analysis. Qual Health Res 2005; 15(9): 1277–88. 16204405 10.1177/1049732305276687

[30] Green BL. Defining trauma: terminology and generic stressor dimensions. J Appl Soc Psychol 1990; 20(20): 1632–42.

[31] Kennedy S, Ruggles S. Breaking up is hard to count: the rise of divorce in the United States, 1980–2010. Demography 2014; 51(2): 587–98. 24399141 10.1007/s13524-013-0270-9 PMC3972308

[32] Crawshaw J, Auyeung V, Norton S, Weinman J. Identifying psychosocial predictors of medication non-adherence following acute coronary syndrome: a systematic review and meta-analysis. J Psychosom Res 2016; 90: 10–32. 27772555 10.1016/j.jpsychores.2016.09.003

[33] Amato PR, Kurian G. The impact of divorce on men and women in India and the United States. Source J Comp Fam Stud 1994; 25(2): 207–21.

[34] Chadda R, Deb K. Indian family systems, collectivistic society and psychotherapy. Indian J Psychiatry 2013; 55(6): 299. 10.4103/0019-5545.105555 PMC3705700 23858272

[35] Carlson EB, Newman E, Daniels JW, Armstrong J, Roth D, Loewenstein R. Distress in response to and perceived usefulness of trauma research interviews. J Trauma Dissociation 2003; 4(2): 131–42.

[36] Mestdagh A, Hansen B. Stigma in patients with schizophrenia receiving community mental health care: a review of qualitative studies. Soc Psychiatry Psychiatr Epidemiol 2014; 49(1): 79–87. 23835576 10.1007/s00127-013-0729-4

[37] Ghooi RB, Deshpande SR. Patients’ rights in India: an ethical perspective. Indian J Med Ethics 2012; 9(4): 277–81. 23099605 10.20529/IJME.2012.092

[38] Raj A, McDougal L. Sexual violence and rape in India. Lancet 2014; 383: 865–6. 10.1016/S0140-6736(14)60435-9 24607092

[39] Sharma R, Pardasani R, Nandram S. The problem of rape in India: a multi-dimensional analysis. Int J Manag Proj Bus 2014; 7(3): 362–79.

[40] Chandra PS, Satyanarayana VA, Carey MP. Women reporting intimate partner violence in India: associations with PTSD and depressive symptoms. Arch Womens Ment Health 2009; 12(4): 203–9. 19283446 10.1007/s00737-009-0065-6 PMC2886198

[41] Zanon C, Hutz CS, Yoo H, Hambleton RK. An application of item response theory to psychological test development. Psicol Refl Crit 2016; 29: 18.

