# Supplementary material for: Adapting the Trauma History Questionnaire for use in a population of homeless people with severe mental illness in Tamil Nadu, India: qualitative study
Source: BJPsych Open. 2021 Jul 5;7(4):e122. doi: 10.1192/bjo.2021.952 (PMC8280791; doi:10.1192/bjo.2021.952)
Supplement: Supplementary file 1 [file S2056472421009522sup001.zip › THQ-MV English Version.pdf]

The Adapted Version of the Trauma-History Questionnaire for Persons with Multiple Vulnerabilities* (THQ-MV) in India

*In this context, multiple vulnerabilities are risk of/homelessness and severe mental illness

About this questionnaire:
The following is a series of questions about serious or shocking life events. Although we like to believe that these kinds of events are rare, they actually happen quite regularly, and can affect how people feel about, react to, and/or think about things afterwards. Knowing about the occurrence of such events and reactions to them, will help us to develop programs to help people who experience these events. The questionnaire is divided into 7 categories of events that you may find distressing: crime-related events, general disasters and traumas, relationship issues, physical and sexual experiences, homeless experiences, (mental) health experiences, and other events.

Instructions:
For each event, please indicate (by circling yes or no) whether it happened, and if it did, the number of times, and around what age you were when it happened. For the items you say yes to, please rate the level of distress it has caused you, from 1 to 5 (1 least distressing and 5 most distressing). Give your best guess if you are not sure. Also please note the nature of your relationship to the person involved and the specific nature of the event, where appropriate. Please answer swiftly and truthfully.

A note to administrator:
This is a newly adapted tool, whose application has only been demonstrated in populations of homeless persons with severe mental illness as of yet. We encourage further adaptation if necessary to fit the specific context in which you wish to administer this tool. We appreciate any feedback on the tool's application. Please contact Andrew Gilmoor (andrewgilmoor@gmail.com) for any correspondence.

Crime related-events

		No	Yes	Number of times	At what age[s]	If Yes, From 1 to 5 please rate the level of distress caused (1 being no distress to 5 being severe distress
1	Has anyone ever tried to take something directly from you (such as property or money) by using force or the threat of force	No	Yes			
2	Has anyone ever tried to rob you or actually robbed you (i.e stole your personal belongings)?	No	Yes			
3	Has anyone ever tried to or succeeded in breaking into your home when you weren't there?	No	Yes			
4	Has anyone ever tried to or succeeded in breaking into your home when you were there?	No	Yes			

General Disasters and Traumas

		No	Yes	Number of times	At what age[s]	If Yes, From 1 to 5 please rate the level of distress caused (1 being no distress to 5 being severe distress
5	Have you ever had a serious accident at work, while commuting (by car, bus, train, etc), or somewhere else? If yes, please specify: ________________ ________________ ________________					
6	Have you ever experienced a natural disaster such as a Cyclone, Tsunami, flood, major earthquake, etc., where you felt you or your loved ones were in danger of death or injury? If yes, please specify if you or your loved one and what event: ________________ ________________ ________________ ________________					

7	Have you ever experienced a human-made disaster such as a train accident, building collapse, fire, etc., where you felt you or your loved ones were in danger of death or injury? If yes, please specify if you or loved one, and what event: ________________ ________________ ________________ ________________					
8	Have you ever been in any other situation in which you were seriously injured? If yes, please specify what type of situation: ________________ ________________ ________________					
9	Have you ever been in any other situation in which you feared you might be killed or seriously injured? If yes, please specify what type of situation: ________________					

	________________<br>________________					
10	Have you ever seen someone seriously injured or killed?<br>If yes, please specify who:<br>________________<br>________________<br>________________					
11	Have you ever seen dead bodies (other than at a funeral) or had to handle dead bodies for any reason?<br>If yes, please specify:<br>________________<br>________________<br>________________					
12	Have you ever had a close friend or family member murdered, or killed by a drunk driver?<br>If yes, please specify who (e.g mother, grandson, etc):<br>________________<br>________________<br>________________					
13	Have you ever had a spouse, romantic partner, parent, or child					

	die?<br>If yes, please specify who:<br>________________<br>________________<br>________________					
14	Have you ever had a spouse, romantic partner, parent, or child commit suicide?<br>If yes, please specify who:<br>________________<br>________________<br>________________					
15	Have you ever had a serious or life threatening illness?<br>If yes, please specify:<br>________________<br>________________					
16	Have you ever witnessed a, friend, relative, parent, romantic partner or spouse suffer from a bad or life threatening illness?<br>If yes, please specify who and what illness:<br>________________<br>________________<br>________________<br>________________					
17	Have you ever received news of a					

	serious injury, life-threatening illness or unexpected death of someone close to you?<br>If yes, please specify who and what happened:<br>________________<br>________________<br>________________<br>________________					
18	Have you ever experienced a sudden financial loss or loss in livelihood, e.g., crops, cattle or fisheries. If yes, please specify what loss:___________<br>________________<br>________________					

Relationship Issues

		No	Yes	Number of times	At what age[s]	If Yes, From 1 to 5 please rate the level of distress caused (1 being no distress to 5 being severe distress
19	Have you ever been the victim of alcohol or substance abuse? If yes, please specify by whom:					

	________________<br>________________					
20	Has shame ever been brought upon your family due to some reason?<br>If yes, please specify what happened:<br>________________<br>________________<br>________________					
21	Have you ever felt alienated or abandoned by a romantic partner, husband, wife, family member, or close friend?<br>If yes, please specify by whom:<br>________________<br>________________<br>________________<br>________________					
22	Has your partner, husband or wife ever had extramarital relations?<br>If yes, please specify with whom:<br>________________<br>________________					
23	Have you ever been separated or divorced?<br>If yes, please specify:					

	________________<br>________________<br>________________<br>________________					
24	Has your partner, husband or wife ever taken up a second spouse?<br>If yes, please specify who:<br>________________<br>________________					

Physical and Sexual Experiences

		No	Yes	Number of times	At what age[s]	If Yes, From 1 to 5 please rate the level of distress caused (1 being no distress to 5 being severe distress
25	Has anyone, including family members or friends, ever attacked you with acid, kerosene, a machete, or some other weapon?<br>If yes, please specify who and with what weapon:<br>________________<br>________________<br>________________<br>________________					
26	Has anyone, including family					

	members or friends, ever attacked you without a weapon?<br>If yes, please specify who:<br>________________<br>________________<br>________________<br>________________					
27	Has anyone in your family ever beaten, slapped or pushed you?<br>If yes, please specify:<br>________________<br>________________<br>________________					
28	Has anyone or a group ever repeatedly bullied, humiliated, tried to intimidate and/or succeeded in intimidating you?<br>If yes, please specify:<br>________________<br>________________<br>________________					
29	Has anyone ever been intimate with you where you felt you couldn't refuse<br>If yes, please indicate nature of relationship with					

	person (e.g stranger, friend, parent, relative, sibling)_________ ________________ ________________					
30	Has anyone ever touched you or made you touch them inappropriately, under force or threat?<br>If yes, please indicate nature of relationship with person (e.g stranger, friend, parent, relative, sibling)_________ ________________ ________________ ________________					

Homeless experiences

		No	Yes	Number of times	At what age[s]	If Yes, From 1 to 5 please rate the level of distress caused (1 being no distress to 5 being severe distress
31	Have you ever experienced a loss of status?					
32	Have you ever gone without					

	shelter? If yes, what was the longest period you’ve gone without shelter?_________					
33	Have you ever gone without food? If yes, what was the longest period you’ve gone without food?___________					
34	Have you ever gone without water? If yes, what was the longest period you’ve gone without water?__________					
35	Have you ever gone without proper hygiene? If yes, what was the longest period you’ve been without proper hygiene?_________					
36	Have you ever been without clothes? If yes, what was the longest period you’ve been without proper clothes?________					
37	Have you ever felt ostracized by society?					

Mental Health Experiences

		No	Yes	Number of times	At what age[s]	If Yes, From 1 to 5 please rate the level of distress caused (1 being no distress to 5 being severe distress
38	Have you ever been troubled or stressed?					
39	Have you ever had a mental condition?					
	If yes, have you ever been stigmatized due to your mental condition?					
40	Have you ever been institutionalized or hospitalized?					
41	Have you ever gone without care for your mental condition. If yes, please specify for how long?__________ _________________					

Other Events

		No	Yes	Number of times	At what age[s]	If Yes, From 1 to 5 please rate the level of distress caused

						(1 being no distress to 5 being severe distress
42	Have you experienced any other extraordinarily stressful situation or event that is not covered above?<br>If yes, please specify:<br>____					
