# Supplementary material for: Adapting the Trauma History Questionnaire for use in a population of homeless people with severe mental illness in Tamil Nadu, India: qualitative study
Source: BJPsych Open. 2021 Jul 5;7(4):e122. doi: 10.1192/bjo.2021.952 (PMC8280791; doi:10.1192/bjo.2021.952)
Supplement: Supplementary file 1 [file S2056472421009522sup001.zip › THQ-MV_Tamil_Version.pdf]

அதிர்ச்சி-வரலாறு சம்மந்தமான தழுவிய பதிப்பு
இந்தியாவில் கடுமையான மன நோயினால் பாதிக்கப்பட்ட வீடற்ற நபர்களை குறித்த வினாத்தாள்

வினாத்தாள் குறித்தவை :

பின்வரும் தொடர் கேள்விகள் கடுமையான அல்லது அதிர்ச்சிகரமான வாழ்க்கையின் நிகழ்வுகள் குறித்தவை. இருந்தபோதிலும் இது போன்ற நிகழ்வுகள் அரிதாக நடக்கிகன்றன என்று நாம் நம்பினாலும், உண்மையில் இது போன்ற நிகழ்வுகள் வழக்கமாக நடந்துகொண்டுதான் இருக்கின்றன. அவை எப்படி மக்களை பாதிக்கின்றன, எப்படி உணர்கின்றனர், எப்படி எதிர்கொள்கிறார்கள் மற்றும்/அல்லது எப்படி அதைப்பற்றி பிறகு சிந்திக்கிறார்கள் என்பதைக் குறித்ததாகும். இது போன்ற நிகழ்வுகளை் மற்றும் அவர்களின் எதிர்வினைகளை அறிந்துக்கொள்வதின் மூலம் இதுபோன்ற நிகழ்வுகளை அனுபவிக்கும் மக்களுக்கு உதவும்படியாக திட்டங்களை உருவாக்க எங்களுக்கு உதவியாயிருக்கும். இந்த வினாத்தாளை 7 நிகழ்வுகளின் வகைகளாக பிரிக்கப்பட்டுள்ள அதன் நோக்கம் தங்களின் துயரத்தை கண்டுபிடித்தல் : குற்றம் தொடர்பான நிகழ்வுகள், பொதுவான பேரழிவுகள், அதிர்ச்சி, உடல் மற்றும் பாலியல் அனுபவங்கள், உறவுமுறை பிரச்சனைகள், வீடற்ற அனுபவங்கள், மன ஆரோக்கிய அனுபவங்கள் மற்றும் மற்ற நிகழ்வுகள்.

வழிமுறைகள்:
கொடுக்கப்பட்டுள்ள ஒவ்வொரு நிகழ்வும் நடந்துள்ளனவா என்பதை அறியும்வண்ணம் தயவுசெய்து (ஆம் அல்லது இல்லை என்பதை வட்டமிடவும்)குறிப்பிடவும். அந்நிகழ்வுகள் நடந்திருந்தால் எத்தனை முறை நடந்ததென்றும் ஏறத்தாழ எந்த வயதில் இது நடந்ததென்று கூறவும். நீங்கள் ஆம் என்று கூறும் கேள்விகளுக்கு தயவுசெய்து உங்களுக்கு ஏற்பட்ட துன்பத்தின் அளவினை 1 முதல் 5 வரையிலுள்ள அளவுகோலில் மதிப்பீடு செய்யவும்.

ஒருவேளை உங்களுக்கு தெரியவில்லையெனில் சிறந்த பதிலை யூகிக்கவும். சம்மந்தப்பட்ட அந்த நபருடன் உங்கள் உறவுமுறையை தெரிவிப்பதோடு அந்த நிகழ்வு குறித்த இயல்பையும் தயவுசெய்து கூறவும்.. உங்கள் பதில்களை துரிதமாகவும் உண்மையாகவும் கூறவும்.

குற்றம் சம்மந்தமான நிகழ்வுகள்

		இல்லை	ஆம்	எத்தனை முறைகள்	எந்த வயதுகளில்	ஆம் என்றால் 1 லிருந்து 5 வரை தயதுசெய்து உங்களுக்கு ஏற்பட்ட துயரத்தின் அளவை மதிப்பிடவும். (1- துயரமில்லை 5 – அதிகமான துயரம்)
1	எப்பொழுதாவது யாரோனும் உங்களை கட்டாயப்படுத்தியோ அல்லது பயமுறுத்தியோ ஏதாவது உங்களிடமிருந்து நேரடியாக எடுத்துக்கொள்ள முயற்சி செய்துள்ளார்களா (சொத்து அல்லது பணம் போன்றவை)	இல்லை	ஆம்			
2	யாராவது எப்போதாவது ஏதேனும் உங்களிடமிருந்து திருட முயன்றுள்ளார்களா ? அல்லது திருடியுள்ளார்களா? (சொந்த உடமைகளை திருடுதல்)	இல்லை	ஆம்			
3	யாராவது எப்போதாவது நீங்கள் உங்கள் வீட்டில் இல்லாதபோது வீட்டை உடைப்பதற்கு முயன்றுள்ளார்களா ? அல்லது உடைத்துள்ளார்களா?	இல்லை	ஆம்			
4	யாராவது எப்போதாவது நீங்கள் உங்கள் வீட்டில் இல்லாதபோது வீட்டை உடைப்பதற்கு முயன்றுள்ளார்களா? அல்லது உடைத்துள்ளார்களா?	இல்லை	ஆம்			

பொதுவான பேரழிவுகள் மற்றும் பேர் அதிர்ச்சிகள்

		இல்லை	ஆம்	எத்தனை முறைகள்	எந்த வயதுகளில்	ஆம் என்றால் 1 லிருந்து 5 வரை தயதுசெய்து உங்களுக்கு ஏற்பட்ட துயரத்தின் அளவை மதிப்பிடவும்.
5	உங்களுக்கு எப்பொழுதாவது பணி இடத்திலோ , பிரயாணத்தின் போது (கார்,பேருந்து, ரயில் வண்டி மற்றவை), அல்லது எங்கேயாவது மிக மோசமான விபத்து ஏற்பட்டுள்ளதா? ஆம் எனில் ... தயவுசெய்து குறிப்பிடவும்: ___ ___	இல்லை	ஆம்			
6	நீங்கள் எப்போதாவது இயற்கை பேரழிவு போன்ற புயல், சுனாமி, வெள்ளம், பயங்கர நில நடுக்கம், மற்றவை ... நீங்கள் அல்லது உங்கள் அன்பானவர்கள் மரண ஆபத்திலோ அல்லது காயப்பட்டுள்ளனர் என்ற உணர்வை நீங்கள் அனுபவத்துள்ளீர்களா? ஆம் எனில், தயவுசெய்து உங்களுக்கோ அல்லது உங்களின் அன்பானவர்களுகக்கோ மற்றும் அது எந்த நிகழ்வு என்று குறிப்பிடவும் : ___ ___	இல்லை	ஆம்			
7	நீங்கள் எப்போதாவது மனிதர்கள் ஏற்படுத்திய பேரழிவாகிய ரயில் விபத்து, கட்டிட இடிந்து விழுதல், தீ விபத்து போன்றவைகளால், அப்போது நீங்கள் அல்லது உங்கள் அன்பானவர்கள் மரன ஆபத்திலோ அல்லது காயம்					

	அடைந்துள்ளார்கள் என்ற உணர்வை அனுபவத்துள்ளீர்களா? ஆம் எனில், தயவுசெய்து உங்களுக்கோ அல்லது உங்களின் அன்பானவர்களுகக்கோ மற்றும் அது எந்த நிகழ்வு என்று குறிப்பிடவும் : ________ ________					
8	நீங்கள் எப்போதாவது மற்ற ஏதாவது சூழ்நிலைகளில் மிக மோசமான காயங்களுக்கு உட்பட்டீர்களா? ஆம் எனில், தயவுசெய்து எந்த வகையான சந்தர்ப்பம் என குறிப்பிடவும்.?					
9	நீங்கள் எப்போதாவது மற்ற ஏதாவது சூழ்நிலையின் காரணமாக நீங்கள் கொலை செய்யப்படுவீர்கள் அல்லது மோசமாக காயப்படுவீர்கள் என்று அச்சப்படும் சூழலில் நீங்கள் எப்போதாவது இருந்ததுண்டா? ஆம் எனில், தயவுசெய்து அது எந்த வகையான சந்தர்ப்பம் என குறிப்பிடவும் ________ ________					
10	நீங்கள் எப்போதாவது யாராவது மிக மோசமான காயங்கள் பட்டோ அல்லது கொல்லப்பட்டவரையோ பார்த்துள்ளீர்களா? ஆம் எனில் தயவுசெய்து யார் என்று குறிப்பிடவும்: ________ ________					
11	நீங்கள் எப்போதாவது இறந்த சடலங்களை பார்துள்ளீர்களா (இறுதி சடங்கை தவிர்த்து) அல்லது இறந்த சடலங்களை ஏதாவது காரணத்திற்காக கையாள நேர்ந்ததுண்டா? ஆம் எனில் தயவுசெய்து அதைப்பற்றி குறிப்பிடவும் ________					

12	நீங்கள் எப்பொழுதாவது உங்கள் நெறுங்கிய நன்பர் அல்லது குடும்ப அங்கத்தினர் கொலை செய்யப்பட்டுள்ளார்கள் அல்லது குடிபோதையிலிருந்த ஓட்டுனரால் விபத்து ஏற்படுத்தி கொலை செய்யப்பட்டுள்ளார்களா?<br>ஆம் எனில்... தயவுசெய்து யார் என்று குறிப்பிடவும் (உ.தா: தாய், பேரன், மற்றவை):<br>____________________<br>____________________<br>____________________					
13	எப்பொழுதாவது நீங்கள் உங்களின் வாழ்க்கைத் துணைவர்/ துணைவி (அ) காதலர், பெற்றோர் அல்லது குழந்தை யாராவது மரணமடைந்துள்ளார்களா?<br>ஆம் எனில், தயவுசெய்து யார் என்று குறிப்பிடவும் .<br>____________________<br>____________________<br>____________________					
14	எப்பொழுதாவது உங்கள் கணவர் வாழ்க்கை துணைவர், துணைவி, பெற்றோர் அல்லது குழந்தை யாராவது தற்கொலை செய்துள்ளார்களா?<br>ஆம் எனில்... தயவுசெய்து யார் என்று குறிப்பிடவும் :<br>____________________<br>____________________<br>____________________					
15	நீங்கள் எப்போதாவது மிக மோசமான அல்லது உயிருக்கு ஆபத்தான நோயினால் பாதிக்கப்பட்டதுண்டா?<br>ஆம் எனில், தயவுசெய்து குறிப்பிடவும்:____________<br>____________________					
16	நீங்கள் எப்போதாவது உங்கள் நண்பர், உறவினர், பெற்றோர் அல்லது கணவன்/மனைவி ஆகியோர் மிக மோசமான					

	அல்லது உயிருக்கு ஆபத்தான வியாதியால் பாதிக்கப்பட்டதை நீங்கள் கண்டதுண்டா?<br>ஆம் எனில், தயவுசெய்து யார் என்றும் என்ன உடல்நல பாதிப்பு என குறிப்பிடவும்:<br>__________<br>__________<br>__________					
17	நீங்கள் எப்போதாவது உங்களுக்கு நெறுங்கியவர்கள் பலத்த காயங்களால், உயிருக்கு ஆபத்தான வியாதிகளால் அல்லது எதிர்பாராத மரண செய்தியை கேட்டதுண்டா?<br>ஆம் எனில், தயவு செய்து யார் என்றும் மற்றும் என்ன நடந்தது என்று குறிப்பிடவும்:<br>__________<br>__________<br>__________					
18	நீங்கள் எப்போதாவது திடீரென பொருளாதார இழப்பு அல்லது வாழ்வாதார இழப்பு, உ தா. பயிர்கள், கால்நடைகள் அல்லது மீன் வளங்களில் இழப்பு ஏற்பட்டுள்ளதை ? அனுபவித்துள்ளீர்களா?<br>ஆம் எனில், தயவு செய்து எவ்வகையான இழப்பு என குறிப்பிடவும்:<br>__________<br>__________<br>__________					

உறவுமுறை பிரச்சனைகள்

		இல்லை	ஆம்	எத்தனை முறைகள்	எந்த வயதுகளில்	ஆம் என்றால் 1 லிருந்து 5 வரை தயதுசெய்து உங்களுக்கு ஏற்பட்ட துயரத்தின் அளவை மதிப்பிடவும்.
19	நீங்கள் எப்போதாவது குடிபழக்கம் அல்லது போதை சம்மந்தமான பழக்கத்தால் அடிமைப்பட்டுள்ளார்களா?<br>ஆம் எனில் தயவுசெய்து யாரால் என்று குறிப்பிடவும்..					
20	உங்கள் குடும்பத்திற்கு எப்போதாவது, ஏதாவது காரணத்திற்காக அவமானம் நேர்ந்ததுண்டா?<br>ஆம் எனில் என்ன நடந்தது என்று குறிப்பிடவும்________________<br>________________________<br>________________________					
21	நீங்கள் எப்போதாவது உங்கள் துணைவர் (அ) துணைவி கணவன், மனைவி குடும்ப உறிப்பினர்கள் (அ) நெருங்கிய நண்பர்களால் ஒதுக்கி வைக்கப்பட்டு (அ) தவிக்கவிடப்பட்டது போன்று உணர்ந்துள்ளீர்களா?<br>ஆம் எனில், யாரால் என்று தயவுசெய்து குறிப்பிடவும்:<br>________________________<br>________________________<br>________________________					
22	உங்கள் துணைவர், கணவன் அல்லது மனைவி எப்போதாவது திருமணத்திற்கு அப்பாறபட்ட உறவில் ஈடுபட்டதுண்டா?<br>ஆம் எனில் தயவு செய்து யாருடன் என்று குறிப்பிடவும்:<br>________________________					

23	நீங்கள் எப்போதாவது பிரிந்தோ அல்லது விவாகரத்து செய்யப்பட்டதுண்டா? ஆம் எனில், தயவுசெய்து யார் என்று குறிப்பிடவும்.					
24	உங்கள் துணைவர், கணவன் அல்லது மனைவி எப்போதாவது இரண்டாவது வாழ்கை துணையை தேர்ந்தெடுத்துள்ளார்களா? ஆம் எனில் தயவுசெய்து யார் என்று குறிப்பிடவும்..					

உடலியல் மற்றும் பாலியல் அனுபவங்கள்

		இல்லை	ஆம்	எத்தனை முறைகள்	எந்த வயதுகளில்	ஆம் என்றால் 1 லிருந்து 5 வரை தயதுசெய்து உங்களுக்கு ஏற்பட்ட துயரத்தின் அளவை மதிப்பிடவும்.
25	யாராவது, உங்கள் குடும்ப அங்கத்தினர் அல்லது நண்பர் உள்பட, எப்போதாவது உங்களை அமிலம், மண்ணெண்னை, வெட்டுகத்தி, வேறு ஏதாவது ஆயுதங்களாலோ உங்களை தாக்கியுள்ளார்களா? ஆம் எனில், தயவு செய்து யார் என்றும் என்ன ஆயுதம் என்று குறிப்பிடவும்: ______ ______ ______ ______					

26	யாராவது , உங்கள் குடும்ப அங்கத்தினர் அல்லது நண்பர் உள்பட எப்போதாவது உங்களை ஆயுதம் இல்லாமல் தாக்கியுள்ளார்களா?<br>ஆம் எனில், தயவு செய்து யார் என்று குறிப்பிடவும் :<br>____________________	இல்லை	ஆம்			
27	உங்கள் குடும்பத்திலுள்ள எவராவது எப்போதாவது உங்களை அடித்தோ அரைந்தோ அல்லது தள்ளியோ உள்ளார்களா?<br>ஆம் எனில், தயவு செய்து யார் குறிப்பிடவும் :<br>____________________<br>____________________	இல்லை	ஆம்			
28	யாராவது உங்களை தனிநபராக அல்லது குழுவாக எப்போதாவது தொடர்ந்து கேளி கிண்டல் அல்லது இழிவு படுத்தியோ அல்லது நெருக்குதலுக்குள்ளாக்க முயற்சியோ அல்லது நெருக்குதலுக்குள்ளாக்கியோ உள்ளார்களா?<br>ஆம் எனில், தயவுசெய்து குறிப்பிடவும்:_______________<br>____________________<br>____________________	இல்லை	ஆம்			
29	யாராவது எப்போதாகிலும் உங்களிடம் நெருக்கமாக இருந்தபொழுது உங்களால் மறுப்பு தெரிவிக்க முடியவில்லை என்று உணர்ந்தீர்களா?<br>ஆம் எனில், அந்த நபருடன் உங்கள் உறவுமுறையை குறிப்பிடவும்(உ.தா அறிமுகம் இல்லாதவர், நண்பர், பெற்றோர், உறவினர்,உடன்பிறப்புக்கள்)_____<br>____________________					
30	யாராவது எப்போதாவது உங்களையோ அல்லது நீங்கள் அவர்களை ~~தகாத இடங்களில்~~					

தேவையில்லாமல்

	தொடும்படியாக உங்களை கட்டாயப்படுத்தியோ அல்லது பயமுறுத்தினார்களா? ஆம் எனில், அந்த நபருடன் உங்கள் உறவுமுறையை குறிப்பிடவும் (உ.தா அறிமுகம் இல்லாதவர், நண்பர், பெற்றோர், உறவினர், உடன் பிறப்புக்கள்____________________ ______________________________					

வீடற்ற அனுபவங்கள்

		இல்லை	ஆம்	எத்தனை முறைகள்	எந்த வயதுகளில்	ஆம் என்றால் 1 லிருந்து 5 வரை தயதுசெய்து உங்களுக்கு ஏற்பட்ட துயரத்தின் அளவை மதிப்பிடவும்.
31	நீங்கள் எப்போதாவது உங்கள் அந்தஸ்தை இழந்ததாக அனுபவித்துள்ளீர்களா?					
32	நீங்கள் எப்போதாவது தங்க இடமின்றி இருந்ததுண்டா? ஆம் எனில், நீங்கள் தங்க இடமின்றி அதிகபட்சமாக எவ்வளவு காலம் இருந்துள்ளீர்கள்? ______________________________					
33	நீங்கள் எப்போதாவது உணவு இல்லாமல் இருந்துள்ளீர்களா? ஆம் எனில், நீங்கள் உணவு இல்லாமல் அதிகபட்சமாக எவ்வளவு காலம் இருந்துள்ளீர்கள்?_______________					
34	நீங்கள் எப்போதாவது தண்ணீர் இல்லாமல் இருந்ததுண்டா? ஆம் எனில், தண்ணீர் இல்லாமல அதிகபட்சமாக எவ்வளவு காலம் இருந்துள்ளீர்கள்?_______________ ______________________________					

35	நீங்கள் எப்பொழுதாவது சரியான சுகாதாரம் இல்லாமல் போனதுண்டா? ஆம் எனில், சுகாதாரம் இல்லாமல் அதிகபட்சமாக எவ்வளவு காலம் இருந்துள்ளீர்கள்? ______					
36	நீங்கள் எப்போதாவது சரியான உடைகள் இல்லாமல் இருந்ததுண்டா ? ஆம் எனில், நீங்கள் சரியான உடைகள் இல்லாமல் அதிகபட்சமாக எவ்வளவு காலம் இருந்துள்ளீர்கள்?______________ ______________________________					
37	நீங்கள் எப்போதாவது சமுதாயத்தால் ஒதுக்கப்பட்டீர்கள் என்று உணர்ந்ததுண்டா?					

மன ஆரோக்கிய சம்மந்தமான அனுபவங்கள்

		இல்லை	ஆம்	எத்தனை முறைகள்	எந்த வயதுகளில்	ஆம் என்றால் 1 லிருந்து 5 வரை தயதுசெய்து உங்களுக்கு ஏற்பட்ட துயரத்தின் அளவை மதிப்பிடவும்.
38	நீங்கள் எப்பொழுதாவது மன நெருக்கடி (அ) மன அழுத்தத்திற்கு ஆளானதுண்டா ?					

39	நீங்கள் எப்போதாவது மன நல பாதிப்பு அடைந்ததுணடா ?<br>ஆம் எனில், எப்போதாவது உங்கள் மன நிலை காரணமாக உங்களுக்கு களங்கம் ஏற்பட்டதுணடா					
40	நீங்கள் எப்போதாவது ஏதாவது நிறுவனத்தில் அல்லது மருத்துவமனையில் அனுமதிக்கப்பட்டதுண்டா?					
41	நீங்கள் எப்பொதாவது உங்கள் மனநிலை சிகிச்சை பெறாமல் சென்றதுண்டா?<br>ஆம் என்றால் தயவுசெய்து எவ்வளவு காலம் என்று குறிப்பிடவும்					

மற்ற நிகழ்வுகள்

		இல்லை	ஆம்	எத்தனை முறைகள்	எந்த வயதுகளில்	ஆம் என்றால் 1 லிருந்து 5 வரை தயதுசெய்து உங்களுக்கு ஏற்பட்ட துயரத்தின் அளவை மதிப்பிடவும்.
42	நீங்கள் எப்போதாவது மேலே கேட்கத் தவறிய ஏதாவது அனுபவங்கள், அசாதாரமான மனஅழுத்தமுள்ள சூழ்நிலையை அல்லது நிகழ்வை அனுபவித்துள்ளீரகளா?..<br>ஆம் எனில், தயவுசெய்து குறிப்படவும்:<br>____________________<br>____________________<br>____________________					
